# Degradation of skeletal mass in locally advanced oesophageal cancer between initial diagnosis and recurrence

**DOI:** 10.1186/s12885-021-09037-3

**Published:** 2021-12-07

**Authors:** Yacine Zouhry, Abdelkader Taibi, Sylvaine Durand-Fontanier, Tiffany Darbas, Geraud Forestier, Jacques Monteil, Valérie Lebrun-Ly, Philippe Fayemendy, Sophie Leobon, Pierre Jesus, Elise Deluche

**Affiliations:** 1 Medical Oncology Department, Limoges University Hospital, 2 avenue Martin Luther King, 87042 Limoges, France; 2 Digestive Surgery Department, Limoges University Hospital, 2 avenue Martin Luther King, 87042 Limoges, France; 3 Neuroradiology Department, Limoges University Hospital, 2 avenue Martin Luther King, 87042 Limoges, France; 4 Nuclear Medicine Department, Limoges University Hospital, 2 avenue Martin Luther King, 87042 Limoges, France; 5 Nutrition Unit, Limoges University Hospital, 2 avenue Martin Luther King, 87042 Limoges, France; 6 Tropical Neuroepidemiology Institute GEIST, INSERM, U1094, 33 rue François Mitterrand, 87032 Limoges, France

**Keywords:** Skeletal muscle mass, Oesophageal cancer, Nutrition, Outcome

## Abstract

**Background:**

The prognostic value of a low skeletal mass index (SMI) has been investigated in locally advanced oesophageal (LAE) cancer at diagnosis. However, nothing is known about its evolution and clinical impact between initial diagnosis and recurrence.

**Methods:**

A total of 89 patients treated for LAE cancer between January 2009 and December 2019 were included in this study. Computed tomography (CT) scans before treatment and at recurrence were evaluated. SMI and other body composition parameters were analysed by the L3 scan method.

**Results:**

Participants were aged 66.0 (36.0–86) years. The incidence of low SMI increased by 12.3% between diagnosis and recurrence (70.7% vs. 83.0%, respectively) over a median follow-up of 16.9 (1.7–101.6) months. Patients with high SMI at diagnosis showed loss of muscle mass (58.0 vs. 55.2 cm^2^/m^2^, respectively; *P* < 0.001) and decreased body mass index (BMI) (27.9 vs. 26.3 kg/m^2^, respectively; *P* = 0.05), but fat mass was increased (68.9 vs. 72.0 cm^2^/m^2^, respectively; *P* = 0.01). Patients with low SMI at diagnosis showed no significant changes in body composition parameters and no improvement of SMI, even with nutritional support. Low SMI (hazard ratio [HR]: 1.8; 95% confidence interval [CI]: 1.02–3.16) was an independent predictor (*P* = 0.041) of high nutritional risk index (HR: 1.79; 95% CI: 1.03–3.11; *P* = 0.039) at diagnosis.

**Conclusions:**

The percentage of patients with a low SMI increased during follow-up. Our data suggest that an assessment of skeletal muscle parameters and nutrition support may be more useful in patients with a high SMI.

**Supplementary Information:**

The online version contains supplementary material available at 10.1186/s12885-021-09037-3.

## Background

Cancer of the oesophagus is the 15th most common cause of cancer in Europe and accounts for 10% of all digestive cancers [1]. The gold standard of treatment for limited disease is endoscopic or surgical resection with neoadjuvant therapy, whereas radiochemotherapy is the gold standard in non-operable and non-resectable cases [2]. Despite some progress in the fields of surgical and medical oncology, the overall 5-year survival rate was slowly improved from 5% in the 1970 s to 20% in 2021. In details, the 5-year survival rate of patients with tumours localised only in the oesophagus is 47%, 25% in cases with disease that has spread to surrounding tissues or organs, and/or regional lymph nodes and 5% if it has spread to distant parts of the body [3]. Several prognostic factors have been studied for oesophageal cancer, including nutrition [4–6] and sarcopenia [7].

Sarcopenia is characterised by a loss of skeletal muscle mass (SMM), skeletal muscle strength and physical performance in quantitative and qualitative terms, as well as in anatomical and functional terms [8]. It was previously shown that assessment of SMM using the cross-sectional area of a single vertebral slice at lumbar L3 thanks computed tomography (CT) scan is well correlated with whole-body skeletal muscle volume. Prado et al. showed for the first time that muscle loss at the start of treatment, as assessed by L3 (CT scan), is a poor prognostic factor for solid tumours [9]. Pre-therapeutic sarcopenia is highly prevalent in cases of cancer and has major implications for patient outcomes [10]. It is important to note that in most studies, sarcopenia is considered to be the same as low skeletal muscle mass (SMM) even though their definition is somewhat different.

Some studies have reported associations between low SMM of patients with oesophageal cancer and oncological outcomes, such as survival [10–12], and suggested that low skeletal muscle mass could be an independent factor associated with pulmonary complications after curative oesophagectomy [11]. ITo our knowledge, most oesophageal cancer studies with longitudinal follow-up have been performed in the context of adjuvant or neoadjuvant therapy [13, 14]. There have been no such studies specifically following up for loss of muscle mass until recurrence. Most oesophageal cancer studies have evaluated patients at diagnosis [7, 10, 11]. One of the issues in the management of cancer patients is how long nutritional management lasts and how long it is effective. Often this is based on weight change alone, but this is likely to be insufficient. We hypothesised that sarcopenia worsens during the management of patients, particularly during a period when patients are less monitored because of post-treatment surveillance. However, we believe that improving sarcopenia is of great importance for patient with recurrent cancer, as they will be more likely to be able to receive adequate treatment for this recurrence[15]. This is why knowing the evolution of sarcopenia is important. Studies in other cancers have shown the importance of this evolution between “primary” and “secondary” sarcopenia [16].

In this study, we evaluated the change of skeletal muscle mass between the time of initial diagnosis and recurrence (local and/or distant) in patients with locally advanced oesophageal cancer. We also assessed the change of other body composition indices and the impact of body composition on morbidity (post-operative complications and length of hospital stay) and survival.

## Methods

### Study population

All consecutive patients treated for locally advanced oesophageal cancer diagnosed at Limoges University Hospital, Limoges, France, between January 2009 and December 2019 were initially included in this study. Among only patients ≥ 18 years, with confirmed diagnosis of locally advanced oesophageal cancer (defined as stage II–III not eligible for primary surgical treatment), and who had a CT scan before treatment (<2 months) and at recurrence were included. Surgical resection of residual tumour after chemoradiotherapy was permitted. The exclusion criteria were the concomitant presence of another neoplasm and a history of another primary cancer within the last 5 years, excluding *in situ* carcinomas of the cervix or previously treated basal cell carcinomas.

### Clinical and biological characteristics

The following data were collected at the time of initial diagnosis and at recurrence: clinical and demographic characteristics, including age, sex, comorbidities (smoking and alcohol consumption) and World Health Organization (WHO) performance status (PS); pathological characteristics, such as TNM stage, histological type and location (classified as cervical and upper, middle, or lower third), surgical characteristics and complications; anthropometric measures of nutritional status, where body mass index (BMI) was used to define undernutrition (BMI < 18.5 kg/m^2^), normal weight (18.5–24.9 kg/m^2^), overweight (25.0–29.9 kg/m^2^) and obesity (≥ 30.0 kg/m^2^) in accordance with the WHO guidelines [17], and weight loss; nutritional management (oral nutritional supplements, enteral nutrition, parenteral nutrition); biological data (C-reactive protein and albumin levels) to calculate the nutritional risk index (NRI; 1.519 × serum albumin level [g/L] + 41.7 × [weight at the beginning of treatment/baseline weight]), where patients were divided into three groups according to NRI score (no-risk group, NRI score > 97.5; moderate risk group, NRI score 97.5–83.5–97.5; severe risk group, NRI score < 83.5) [18]; and treatment characteristics, including chemotherapy protocol and radiotherapy data (total dose and treatment duration).

### Analyses of body composition by CT

Digital images were retrieved from the Picture Archiving and Communication System (Telemis version 4.7; Telemis SA, Louvain la Neuve, Belgium). All images were reviewed and analysed by a radiologist blinded to patient survival status using AW software (GE Healthcare, Waukesha, WI, USA), as described previously [19]. Skeletal muscle mass, visceral adipose tissue (VAT), subcutaneous adipose tissue (SAT) and infiltration inter-muscular adipose tissue (IMAT) were identified and quantified from a single image at the third lumbar vertebra (L3) using the following Hounsfield unit (HU) thresholds: muscle, −29 to 150 HU; VAT, −150 to −50 HU; SAT and IMAT, −190 to −30 HU. The skeletal mass index (SMI), VAT index, SAT index and IMAT index were calculated in cm^2^/m^2^ by tissue cross-sectional area (cm^2^) normalised to square metres (m^2^).

Skeletal muscle mass (skeletal muscle index, cm^2^/m^2^) and density (HU) at the level of the third lumbar vertebra were measured on contrast-enhanced CT images.

The estimated skeletal muscle mass and density for each subject, and the lower bound of the 90% prediction interval, were calculated.

The cut-offs for SMI were 38.5 cm2/m2 for females and 52.4 cm2/m2 for males [9, 20]. Females SMI < 38.5 cm2/m2and men with SMI 52.4 cm2/m2 were considered to have low SMI.

### Endpoints of the study

The primary endpoint was the change of SMI between diagnosis and recurrence in patients with locally advanced oesophageal cancer.

The secondary endpoints were the change of SMI between diagnosis and recurrence according to the initial SMI, the change of other body composition parameters (VAT index, SAT index and IMAT index) and BMI status between initial diagnosis and recurrence, in the whole cohort and according to the initial SMI, and survival according to SMI (evaluated at diagnosis and recurrence).

### Statistical analysis

Quantitative results are expressed as the mean ± standard deviation (SD) or median (range), and qualitative results are expressed as numbers and percentages. Nominal variables were compared between the groups using the chi-square or Fisher’s exact test, as appropriate. Means were compared using the non-parametric Mann–Whitney *U* test for continuous variables and the Wilcoxon signed-rank test for paired tests. The paired *t* test was used to examine correlations of changes between diagnosis and recurrence with changes in BMI, SMI and body composition.

Disease-free survival (DFS) was defined as the interval between surgery and the date of first recurrence. Overall survival (OS) was defined as the time between the date of diagnosis of metastatic disease and the date of death (any cause) or censored to the date of last contact. We used the Kaplan–Meier method to estimate OS and DFS, and the log-rank test to assess differences between SMI subgroups. The Cox proportional hazards model was used to identify prognostic factors of OS and DFS in the whole cohort, and to calculate hazard ratios (HRs) and 95% confidence intervals (CIs). Variables with a *P*-value < 0.10 in univariate analyses were included in multivariate analyses. The variables included in the model were age at diagnosis (< or ≥ 65 years), NRI (≤ or > 97.5), first-line chemotherapy (yes or no) and SMI (low or high). *P* < 0.05 was taken to indicate statistical significance. All data were analysed using StatView software (SAS Institute, Cary, NC, USA) and R software (v.3.5.1; The R Foundation for Statistical Computing, Vienna, Austria).

### Ethical approval

 Clinical data were collected in accordance with French bioethics laws regarding patient information and consent. Data collection and use were approved by the Limoges Hospital Ethics Committee (President, Dr. Terrier) (approval number 370–2020–26). All patients provided written informed consent for the collection and use of data from biological samples at the beginning of medical care. The use of retrospective and prospective data from the BRTS (regional solid tumour base) was also approved by the Ethics Committee (approval number 200–2016–14).

## Results

### Patient characteristics at baseline and recurrence

Among the total of 91 patients, baseline data were available for 89 and these patients constituted the whole cohort (eFigure 1). Tables 1 and 2 summarise the patient and tumour characteristics of the study cohort at diagnosis (*n* = 89) and recurrence (*n* = 47).

**Table 1:** Patient and tumour characteristics at diagnosis in all cohorts and according to skeletal mass index

Clinical and pathological parameters	Total	High SMI	Low SMI	*P*-value
	*n* = 89	*n* = 26 (29.1%)	*n* = 63 (70.7%)	
**Age** (years), median (range)	66.0 (36.0–86.0)	64.0 (36.0–78.0)	67.0 (42.0–86.0)	0.08
Sex				
Male/female	73 (82.0)/16 (18.0)	22 (15.4)/4 (84.6)	51 (19.0)/12 (81.0)	0.6
Smoking status				0.2
Yes	33 (37.0)	10 (38.5)	23 (36.5)	
No	13 (14.6)	6 (23.0)	7 (11.1)	
Ex-smoker	43 (48.4)	10 (38.5)	33 (52.4)	
Alcohol drinker				0.1
Yes	29 (32.5)	10 (38.5)	19 (30.1)	
No	13 (14.6)	6 (23.0)	7 (11.1)	
Ex-alcohol drinker	47 (52.9)	10 (38.5)	37 (58.8)	
Tumour histological subtype				0.03
Squamous cell carcinoma	53 (59.6)	11 (42.3)	42 (66.6)	
Adenocarcinoma	36 (40.4)	15 (57.7)	21 (33.4)	
Endobrachyoesophageal status				0.4
Yes/No	13 (14.6)/76 (85.4)	5 (19.2)/21 (80.8)	8 (12.7)/55 (87.3)	
TNM Stage				0.1
II	42 (47.2)	9 (34.6)	33 (52.4)	
III	47 (52.8)	17 (65.4)	30 (47.6)	
Tumour location				0.4
Upper	20 (22.4)	4 (15.4)	16 (25.4)	
Mean	26 (29.3)	6 (23.0)	20 (31.7)	
Lower	38 (42.7)	14 (53.9)	24 (38.1)	
More than two locations	5 (5.6)	2 (7.7)	3 (4.8)	
Performance status				0.006
0/1	74 (83.2)	26 (100.0)	48 (76.2)	
2/3	15 (16.8)	0 (0)	15 (23.8)	
Nutritional parameters				
BMI (kg/m^2^) before the cancer diagnosis				
Median (range)	26.3 (17.0–52.9)	28.8 (21.2–52.9)	25.1 (17.0–37.6)	0.0001
Underweight (< 18.5)	1 (1.1)	0 (0)	1 (1.6)	0.006
Normal (≥ 18.5 < 25.0)	31 (34.8)	3 (11.5)	28 (44.4)	
Overweight (≥ 25.0 < 30.0)	18 (20.2)	10 (38.5)	8 (12.7)	
Obese (≥ 30.0)	39 (43.9)	13 (50)	26 (41.3)	
NRI at cancer diagnosis				0.6
> 97.5	26 (29.2)	7 (27.0)	19 (30.2)	
97.5–83.5	41 (46.0)	11 (42.3)	30 (47.6)	
< 83.5	22 (24.8)	8 (30.7)	19 (22.2)	
Albumin level (g/L) median (range)	36.0 (17.5–48.0)	35.5(22.0–43.0)	36.2(17.5–48.0)	0.3
C-reactive protein, median (range)	9.5(1.0–113.0)	9.0(1.0–96.0)	12(1.0–113.0)	0.9
BMI (kg/m^2^) at cancer diagnosis				
Median (range)	24.1 (14.9–47.7)	27.9 (20.9–47.7)	22.6(14.9–37.2)	< 0.0001
Underweight (< 18.5)	10 (11.1)	0 (0)	10 (15.9)	0.0008
Normal (≥ 18.5 < 25.0)	40 (44.9)	8 (30.7)	32 (50.8)	
Overweight (≥ 25.0 < 30.0)	30 (33.8)	11 (42.3)	19 (30.2)	
Obese (≥ 30.0)	9 (10.1)	7 (27.0)	2 (3.1)	
Weight loss (%) median (range)	8.0 (– 1.2–38.7)	6.5 (0–27.0)	9.0 (– 1.2–38.7)	0.2
Special nutritional management				0.4
Oral nutritional supplements	19 (21.3)	7 (27.0)	12 (19.0)	
Enteral nutrition	66 (74.1)	17 (65.3)	49 (77.7)	
Parenteral nutrition + enteral nutrition	4 (4.5)	2 (7.7)	2 (3.1)	
Current therapies, *n* (%)				0.3
Chemotherapy	22 (24.7)	8 (30.7)	14 (22.2)	
Radiotherapy	10 (11.2)	1 (3.9)	9 (14.3)	
Radiochemotherapy	57 (64.1)	17 (65.4)	40 (63.5)	
Surgery (yes)	19 (21.4)	8 (30.7)	11 (17.4)	0.1
Complications	14 (73.6)	6 (75.0)	8 (72.7)	0.9
Respiratory complications	10 (52.6)	5 (62.5)	5 (45.4)	0.4
Length of hospital stay (days)	28.0 (14.0–60.0)	24.0 (14.0–44.0)	29.5 (19.0–60.0)	0.4
Relapse				0.04
No	42 (47.2)	12 (46.1)	30 (47.6)	
Local relapse	23 (25.8)	3 (11.5)	20 (31.7)	
Metastatic relapse	24 (27.0)	11 (42.3)	13 (20.6)	

BMI: body mass index; NRI: nutritional risk index; SMI: skeletal muscle index

**Table 2:** Patient and tumour characteristics at recurrence in all cohorts and according to skeletal mass index

	Total at relapse	High SMId-High SMIr	High SMId-low SMIr	Low SMId-Low SMIr	Low SMId-High SMIr
	*n* = 47	*n* = 4	*n* = 10	*n* = 29	*n* = 4
Age (years), median (range)	66.0 (36.0–84.0)	74.0 (63.0–79.0)	62.0 (37.0–69.0)	68.0 (43.0–85.0)	72.0 (67.0–77.0)
Sex					
Male/female	39 (83.0)/8 (17.0)	2(50)/2(50)	0 (0)/10 (100)	5 ()/24 ()	1 ()/3 ()
Performance status					
0/1	37 (78.7)	4 (100)	10 (100)	22 (75.8)	3 (75)
2/3	10 (21.2)	0 (0)	0 (0)	7 (24.2)	1 (25)
NRI*					
> 97.5	7 (17.0)	1 (25)	0 (0)	6 (24.0)	0 (0)
97.5–83.5	18 (44.0)	2 (50)	3 (33.4)	8 (32.0)	2 (66.6)
< 83.5	16 (39.0)	1 (25)	6 (66.6)	11 (44.0)	1 (33.4)
Albumin levels (g/L)*	34.0 (18.0–44.5)	34.8 (22.7–40.2)	26.5 (20.2–40.0)	35.0 (18.4–44.5)	35.6 (18.0–39.0)
C-reactive protein**	14.0 (1.0–254.0)	8.5 (3.0–14.0)	26.0 (1.0–199.0)	13.0 (1.0–178.0)	20.5 (3.0–254.0)
BMI (kg/m^2^)					
Median (min–max)	23.8 (16.4–34.6)	30.3 (26.1–34.6)	24.6 (19.7–29.8)	23.1 (16.4–30.8)	24.9 (21.7–26.6)
Underweight (< 18.5)	5 (10.6)	0 (0)	0 (0)	5 (17.2)	0 (0)
Normal (≥ 18.5 < 25.0)	22 (46.8)	0 (0)	6 (60)	14 (48.2)	2 (50)
Overweight (≥ 25.0 < 30.0)	3 (6.4)	2 (50)	0 (0)	1 (3.4)	0 (0)
Obese (≥ 30.0)	17 (36.2)	2 (50)	4 (40)	9 (31.0)	2 (50)
Special nutritional management***					
Oral nutritional supplements	22 (48.9)	1 (25)	5 (55.5)	13 (46.4)	3 (75)
Enteral nutrition	21 (46.7)	3 (75)	3 (33.3)	14 (50)	1 (25)
Parenteral nutrition	2 (4.4)	0 (0)	1 (11.2)	1 (3.7)	0 (0)

*6 missing data

**10 missing data

***2 missing data

BMI: body mass index; d: at diagnosis, r: at relapse; NRI: nutritional risk index; SMI: skeletal muscle index

The median ages of the patients at initial cancer diagnosis and recurrence were 66.0 (36.0–86.0) and 66.0 (36.0–84.0) years, respectively. The median BMI were 26.3 (17.0–52.9), 24.1 (14.9–47.7) and 23.8 (16.4–34.6) kg/m^2^ before diagnosis, at diagnosis of cancer and at recurrence, respectively. At diagnosis, 10 patients (11.1%) were undernourished, 40 (44.9%) were normal weight, 30 (33.8%) were overweight and 9 (10.1%) were obese. The weight loss difference between before diagnosis and at diagnosis was −8.0% [−1.2, −38.7], and that between diagnosis and recurrence was −3.1% [−27.9, 28.1].

All patients were on nutritional management and had access to personalised dietary counselling. There were no differences in nutritional management between the two groups (*P* = 0.4) (Tables 1 and 2).

Patients received these regimens of chemotherapy : FOLFOX, EOX or cisplatin. The major surgical procedure performed was a Lewis-Santy oesophagectomy. The median dose of radiotherapy was 50.4 Gy (50-60 Gy).

### Changes in SMI and body composition parameters during follow-up

In the whole cohort, the proportion of patients with a low SMI was 70.7% at diagnosis and 83.0% at recurrence, representing an increase of 12.3%. At recurrence, only four patients maintained a high SMI, while 10 maintained a low one. Nineteen patients remained at a low SMI and four changed to a high SMI (Table 3). The rates of SMI are shown in Table 3.

**Table 3:** Changes of skeletal muscle index between diagnosis and recurrence

At initial diagnosis	At relapse		Total
	High SMI	Low SMI	
High SMI	*n* = 4 SMI at diagnosis: 58.0 (42.6–75.2) SMI at relapse: 52.8 (39.4–78.2))	*n* = 10 SMI at diagnosis: 58.2 (53.0–62.6) SMI at relapse: 47.7 (41.6–52.3)	14
Low SMI	*n* = 4 SMI at diagnosis: 44.9 (35.8–51.0) SMI at relapse: 55.3 (41.3–61.3)	*n* = 29 SMI at diagnosis: 42.8 (32.8–51.8) SMI at relapse: 42.8 (30.7–52.1)	33
Total	8	39	47

SMI: skeletal muscle index

SAT and IMAT remained stable. VAT increased between diagnosis and recurrence (52.2 and 58.6 cm^2^/m^2^, respectively; *P* < 0.001), whereas BMI decreased during the follow-up period (24.1 and 23.8 kg/m^2^, respectively; *P* = 0.04) (Table 4).

**Table 4:** Changes in body composition between diagnosis and recurrence

	Whole cohort			Initial high SMI group			Initial low SMI group		
	At diagnosis	At relapse	*P*-value*	At diagnosis	At relapse	*P*-value*	At diagnosis	At relapse	*P*-value^a^
SMI (cm^2^/m^2^), median (range)	44.9 (25.3–75.2)	45.4 (30.8–78.2)	0.08	58.0 (42.6–75.2)	55.2 (39.4–78.2)	< 0.001	42.3 (25.3–51.8)	44.5 (30.8–52.3)	0.71
VAT index (cm^2^/m^2^), median (range)	52.2 (1.4–164.4)	58.6 (3.7–160.7)	< 0.001	68.9 (6.9–159.8)	72.0 (16.4–95.6)	0.014	43.7 (1.4–164.4)	56.7 (3.7–160.7)	0.82
SAT index (cm^2^/m^2^), median (range)	44.5 (0.6–61.8)	43.7 (5.09–198.4)	0.8	23.6 (2.4–55.3)	71.1 (21.2–198.4)	0.77	15.6 (0.6–61.8)	42.7 (5.09–101.9)	0.54
IMAT index (cm^2^/m^2^), median (range)	0.12 (0.0–0.5)	0.09 (0.02–0.5)	0.9	0.09 (0.1–0.5)	0.09 (0.02–0.5)	0.60	0.11 (0.0–0.5)	0.10 (0.02–0.4)	0.74
BMI (kg/m^2^)	24.1 (14.9–47.7)	23.8 (16.4–34.6)	0.04	27.9 (20.9–47.7)	26.3 (21.7–34.6)	0.05	22.6 (14.9–37.2)	23.5 (16.4–30.8)	0.21

IMAT: infiltration inter-muscular adipose tissue; SAT: subcutaneous adipose tissue; SMI: skeletal muscle index; VAT: visceral adipose tissue

^a^paired *t* test

### Patient characteristics and body composition parameters according to SMI

The characteristics of the patients according to SMI (high vs. low), at diagnosis and at recurrence, are listed in Table 1 and 2. Significant differences in PS scores were observed between the groups, with 100% of high SMI patients having a “good” PS score compared to 76.2% of low SMI patients (*P* = 0.006).

In the low SMI group at diagnosis, all body composition parameters remained stable between diagnosis and recurrence (Table 4). Between diagnosis and recurrence, the group with a high SMI at diagnosis showed significant decreases in muscle mass (58.0 and 55.2 cm^2^/m^2^, respectively; *P* < 0.001) and BMI (27.9 and 26.3 kg/m^2^, respectively; *P* = 0.05), whereas their fat mass increased significantly (68.9 and 72.0 cm^2^/m^2^, respectively; *P* = 0.01) (Table 4).

### Multivariate analyses of DFS and OS according to SMI at diagnosis

The median follow-up of the cohort was 16.9 (1.7–101.6) months. OS and DFS were assessed according to SMI at diagnosis.

**Fig. 1:**
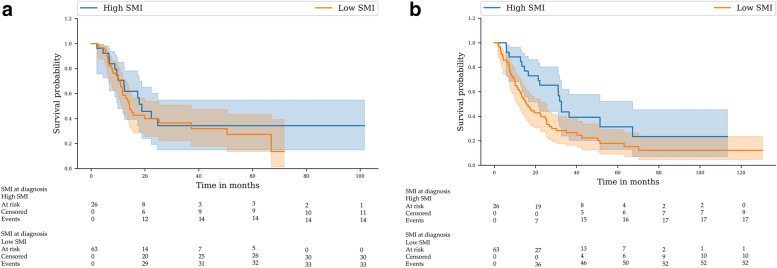
(**a**) Disease-free survival in the two skeletal muscle index groups. (**b**) Overall survival in the two skeletal muscle index groups Blue: high skeletal mass index (SMI); red: low skeletal mass index (SMI)

Among patients with a low SMI at diagnosis, 33 (52%) showed disease recurrence after treatment, with a median time to recurrence of 14.2 months, whereas 14 (53%) patients with a high SMI showed disease recurrence after a median of 18.8 months. In univariate analysis, no differences in DFS were observed between the two groups (Fig. 1a) (*P* = 0.51).

The median OS of the cohort was 21.3 (95% CI: 14.8–27.9) months. Fifty-two patients (82.5%) in the low SMI group died compared to 17 (65.3%) in the high SMI group.

As shown in Fig. 1b, the median OS was 15.8 (95% CI: 12.2–25.3) months in the low SMI group and 31.7 (95% CI: 22.1–NA) months in the high SMI group (*P* = 0.023). In multivariate analyses, a low SMI (HR: 1.80; 95% CI: 1.02–3.16; *P* = 0.0417) and NRI ≤ 97.5 (HR: 1.79; 95% CI: 1.03–3.11; *P* = 0.04) were positively associated with mortality risk (Table 5).

**Table 5:** Univariate and multivariate analyses of body composition parameters associated with overall survival

	Overall survival			
	Univariate		Multivariate	
	HR (95% CI)	*P*-value	HR (95% CI)	*P*-value
Age at diagnosis (< 65 years)	0.5953 [0.364–0.9736]	0.0388	0.6853 [0.4089–1.1485]	0.1515
NRI (≤ 97.5)	1.6996 [0.9797–2.949]	0.0592	1.7865 [1.0270–3.1080]	0.0399
Chemotherapy (yes)	0.4667 [0.2265–0.9617]	0.0388	0.6122 [0.2840–1.3197]	0.2105
Low SMI	1.8830 [1.084–3.272]	0.0248	1.7973 [1.0224–3.1590]	0.0417

NRI: nutritional risk index; SMI: skeletal muscle index

### Multivariate analyses of DFS and OS according to SMI at recurrence

OS and DFS were assessed according to SMI at recurrence. No differences in DFS were observed between the groups over time, including after analysing the variation in SMI during follow-up (eFigure 2). SMI was not an independent negative predictor of OS in multivariate analyses.

## Discussion

This study was performed to examine whether baseline skeletal muscle mass indices change over the follow-up period in patients with locally advanced oesophageal cancer. To our knowledge, this is the first study to demonstrate a change in SMI in this population between diagnosis and recurrence. We showed an increase of 12.3% in the low SMI group, indicating degradation of skeletal muscle mass.

The prevalence of a low SMI at diagnosis in the present study was 70.7%, consistent with previously reported rates of 25.5–86.0% [21–32] and validating our initial population selection. The prevalence of a low SMI increased to 83.0% at recurrence. Body composition remained stable in the low SMI group, whereas the high SMI group showed a decrease in muscle mass and increase in fat mass. This decrease in skeletal muscle mass was greater than would be expected naturally (approximately 1.4% per year due to physiological aging) [33].

Few studies have evaluated changes in body composition during follow-up of oesophageal cancer patients, and data have only been collected in the context of adjuvant and neoadjuvant therapy [13, 14]. The prevalence of sarcopenia in oesophageal cancer was reported to increase during neoadjuvant treatment [34], consistent with our findings. Regarding other types of cancer, two studies observed long-term loss of SMI in patients with medullary thyroid carcinoma or renal carcinoma receiving treatment (6–9 months) [35, 36], while an adjuvant breast cancer study observed an increase in total body fat and decreases in fat-free mass and lean soft tissue, but no changes in body weight [37].

One possible explanation for the increased prevalence of low SMI, even with nutritional support, is a lack of exercise training to enhance muscle strength and function. Sarcopenia has been a topic of recent interest in the context of physical activity. It would be useful to obtain data on changes of muscle strength (e.g., results of the handgrip test) in these patients, particularly those who have maintained or gained lean mass. Exercise rehabilitation has been investigated in cancer patients, including those with breast cancer, and was shown to have a positive effect on muscle strength [38, 39]; similar findings were also reported in oesophago-gastric cancer survivors [40]. However, the RESTORE program (exercise training, dietary counselling and multidisciplinary education) showed that body composition remained stable even after targeted training [41]. Similarly, Guinan et al. reported that despite maintenance of functional capacity and activities, the muscle mass and strength of patients (*n* = 28) declined between pre- and post-neoadjuvant therapy [42].

In addition to various factors such as age, PS, disease stage and comorbidities, a low SMI could be another useful parameter to incorporate into the clinical decision-making process. The present study showed that a low SMI at diagnosis was an independent prognostic factor. Three large systematic reviews and meta-analyses of pre-operative sarcopenia in patients with oesophageal cancer concluded that sarcopenia is associated with poorer OS, as determined by CT in patients from European centres, regardless of whether the patients received pre-operative treatment or the definition of sarcopenia was used [7, 10, 11]. A systematic review and meta-analysis reported a significant increase in overall morbidity (relative risk [RR]: 1.16; 95% CI: 1.01–1.33) in patients with sarcopenia [43], and another study reported poorer long-term outcomes after oesophagectomy in sarcopenic patients (HR: 1.70; 95% CI: 1.33–2.17) [7]. Deng et al. reported that patients with sarcopenia had significantly lower 3-year (51.6% and 65.4%, respectively) and 5-year OS rates (41.2% and 52.2%, respectively) than those without sarcopenia [11].

The present study could not conclusively determine the role of sarcopenia in post-operative complications but it was not the main scope of this project. Most previous studies reported that pre-operative sarcopenia, as assessed by CT, was not associated with significantly higher rates of overall post-operative complications or overall mortality [7, 10, 11]. More precisely, an association between pre-operative sarcopenia and post-oesophagectomy pulmonary complications has been reported; a higher incidence of post-operative pulmonary complications (RR: 2.03; 95% CI: 1.32–3.11, *P* = 0.001) was observed in sarcopenia patients after oesophagectomy [7], while Papaconstantinou et al. observed an increase in the rate of respiratory complications (RR: 1.64; 95% CI: 1.21–2.22) among such patients [43]. However, a recent meta-analysis of eight studies involving 1,488 patients suggested that sarcopenia does not affect the rate of post-operative complications, including respiratory complications, in patients undergoing oesophagectomy for oesophageal cancer [44].

The last question we aimed to answer was whether it is necessary to monitor the SMI [45]. We showed that only the initial SMI had an impact on outcome [7, 10, 11]. CT is often used to evaluate the whole body before treatment; measuring the SMI using this modality is clinically convenient, with no requirement to determine changes in SMI during follow-up. Although body mass index at the time of recurrence was not associated with survival outcomes in our patients, Measuring grip strength or walk test is simple and inexpensive and may be relevant for early detection of sarcopenia and modification of our management [46].

This study had some limitations. First, it was a retrospective investigation conducted at a single institution. One of the limitations of this retrospective study is the heterogeneity of management between 2009 and 2019 due to the variation in recommendations. These data could better analyzed on a prospective clinical trial. Second, we evaluated only low SMI and not sarcopenia, as defined by the European Working Group on Sarcopenia in Older People (EWGSOP), because of the nature of the study [8]. However, this study fits perfectly with a multidisciplinary healthcare approach, in which screening for low skeletal mass should involve a nutritionist once CT has been performed. Third, the cut-off values for diagnosis of sarcopenia remain controversial and could vary according to the population of interest. The Asian Working Group for Sarcopenia (AWGS) published specific consensus guidelines to define sarcopenia in this population [47, 48]. In this study, we used the cut-off proposed by Prado et al. [9]. However, no cut-off value has been validated in a prospective study, particularly for oesophageal cancer. Due to the confusion between sarcopenia and low SMI in the initial studies, the most frequently used cut-offs historically are those of Prado et al. who initially referred to sarcopenia and not SMI.

## Conclusion

At diagnosis, SMI was an independent prognostic factor in patients with local advanced oesophageal cancer. Low SMI was shown to be associated with poor survival outcomes at diagnosis. Evaluation of SMI at recurrence may not change the outcome of oesophageal cancer. It appears essential to accurately evaluate sarcopenia to develop multimodal interventions integrating nutritional support and physical exercise specifically for oesophageal cancer patients, to improve muscle mass and function. The change of SMI emphasises the need to increase nutritional intake and physical activity in this group of patients over the long term. Today, the methods for detecting sarcopenia are improving, but we do not yet know how to correct it in order to improve patient care.

## Supplementary Information

**Additional file 1: eFigure 1.** Study flowchart.

**Additional file 2: eFigure 2.** a) Disease-free survival in the two skeletal muscle index groups at relapse. b) Overall survival in the two skeletal muscle index groups at relapse.

## Data Availability

Data are available from the corresponding author upon reasonable.

## Abbreviations

BMI: body mass index
CI: confidence interval
CT: computed tomography
DFS: disease-free survival
HR: hazard ratio
HU: Hounsfield units
IMAT: infiltration inter-muscular adipose tissue
NRI: nutritional risk index
OS: overall survival
PS: performance status
SAT: subcutaneous adipose tissue
SMI: skeletal muscle index
VAT: visceral adipose tissue
