# Supplementary figures and images for: Degradation of skeletal mass in locally advanced oesophageal cancer between initial diagnosis and recurrence
Source: BMC Cancer. 2021 Dec 7;21:1313. doi: 10.1186/s12885-021-09037-3 (PMC8653570; doi:10.1186/s12885-021-09037-3)

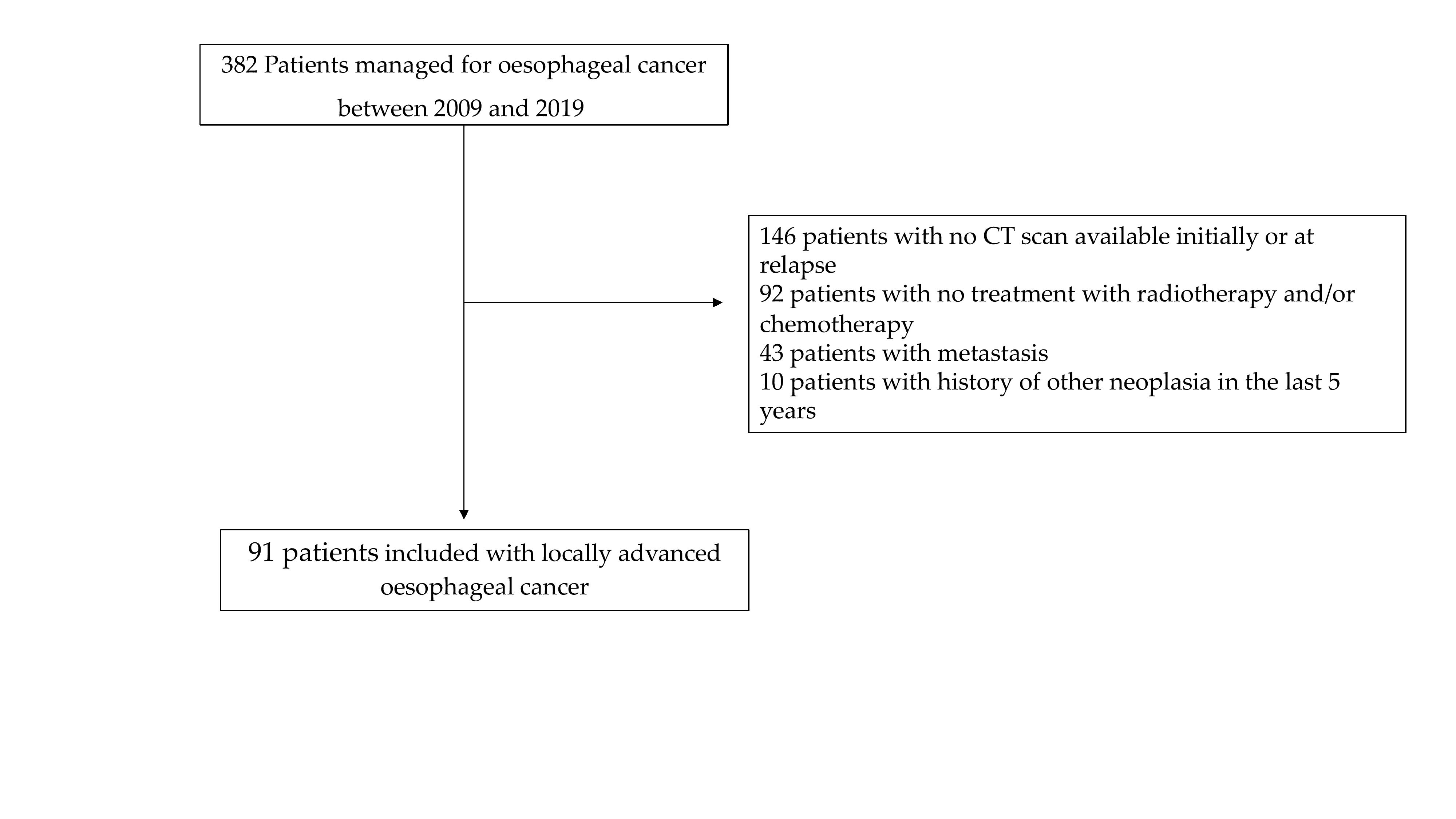

Supplement: Supplementary file 1 — Additional file 1: eFigure 1. Study flowchart. [file 12885_2021_9037_MOESM1_ESM.jpg]

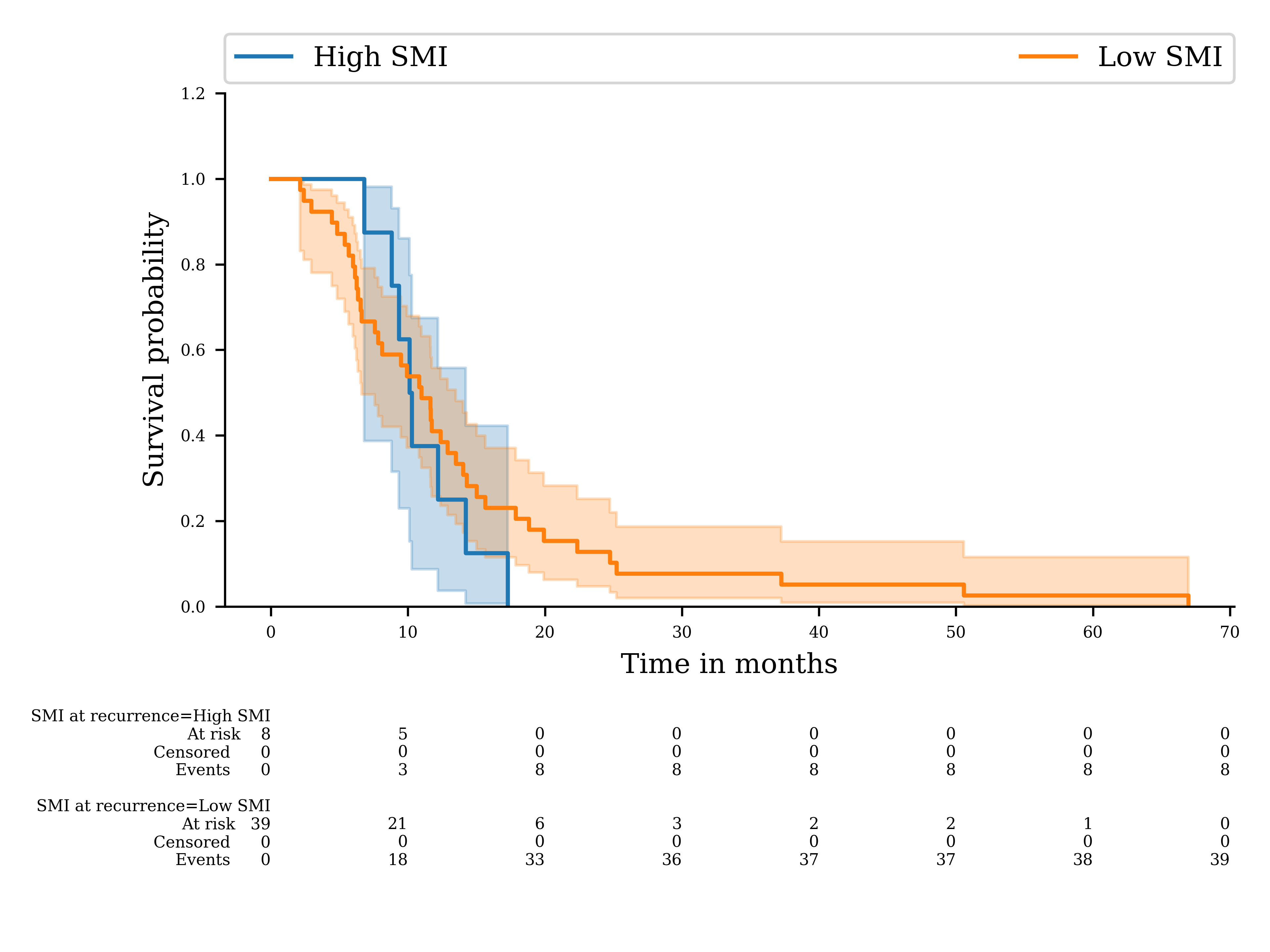

Supplement: Supplementary file 2 — Additional file 2: eFigure 2. a) Disease-free survival in the two skeletal muscle index groups at relapse. b) Overall survival in the two skeletal muscle index groups at relapse. [file 12885_2021_9037_MOESM2_ESM.zip › eFigure 2a review.png]

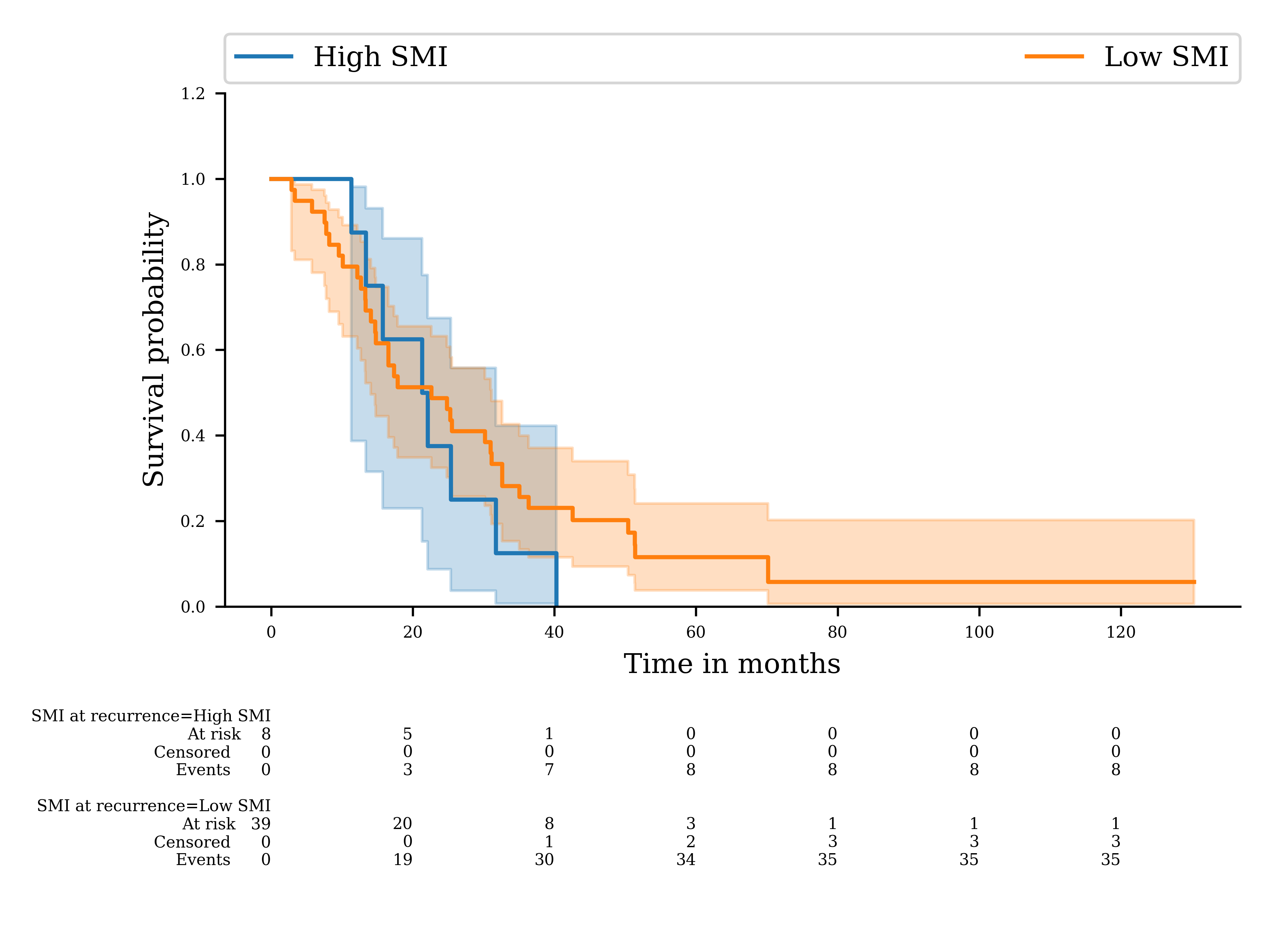

Supplement: Supplementary file 2 — Additional file 2: eFigure 2. a) Disease-free survival in the two skeletal muscle index groups at relapse. b) Overall survival in the two skeletal muscle index groups at relapse. [file 12885_2021_9037_MOESM2_ESM.zip › eFigure 2b review.png]
